# Study on the noncoincidence effect phenomenon using matrix isolated Raman spectra and the proposed structural organization model of acetone in condense phase

**DOI:** 10.1038/srep43835

**Published:** 2017-03-03

**Authors:** Wenwen Xu, Fengqi Wu, Yanying Zhao, Ran Zhou, Huigang Wang, Xuming Zheng, Bukuo Ni

**Affiliations:** 1 Department of Chemistry and Engineering Research Center for Eco-dyeing and Finishing of Textiles, MOE, Zhejiang Sci-Tech University, Hangzhou 310018, China; 2 Texas A&M Univ, Dept Chem, Commerce, TX 75429, USA

## Abstract

The isotropic and anisotropic Raman spectra of acetone and deuterated acetone isolated in an argon matrix have been recorded for the understanding of noncoincidence effect (NCE) phenomenon. According to the matrix isolated Raman spectra and DFT calculations, we proposed aggregated model for the explanations of the acetone C=O vibration NCE phenomenon and its concentration effect. The experimental data were in consistence with the DFT calculations performed at the B3LYP-D3/6-311 G (d,p) levels based on the proposed model. The experimental identification of the monomer, dimer and trimer are reported here, and the dynamic of the transformation from monomer to aggregated structure can be easily controlled by tuning annealing temperature.

Carbonyl compounds are engaged in a wide variety of biological processes and play important roles in industry and life activities^1^,^2^,^3^. Many living systems are based on the structure and dynamics of carbonyl based clusterized biomolecules^4^,^5^,^6^. The photosignal transduction in *Natronobacterium pharaonis* is dependent on the dynamic transition of complex structure of Sensory SRII and transducer HtrII (NpSRII/NpHtrII) which is clusterized by hydrogen bond and van der Waals force^6^. It is well known that the C=O stretching frequency is sensitive to the environmental change and consequently allows the investigation of the secondary structure of proteins at different environment^7^,^8^,^9^,^10^. Mizuno M. etc.^7^,^8^ employed time-resolved Raman spectroscopy by detecting spectral changes in the tryptophan and tyrosine bands to probe protein structure changes. C=O stretching frequency can also be utilized as a sensitive probe for the investigation of the solute structure and the solution environment changes. It has been intensively studied for noncoincidence effect (NCE) phenomenon that is first and most remarkably observed in carbonyl compounds^11^,^12^,^13^,^14^, the mostly acceptable mechanism for NCE is transition dipole-transition dipole (td-td) coupling theory interactions in a short range or long-range orientational order of molecular dipoles^15^,^16^,^17^. Acetone is a model molecules for investigation of noncoincidence effect and concentration dependent effect^15^. Nearly half a century ago, the shift in the C=O Raman wavenumber in different concentration was reported^18^. Positive NCE and negative ion-perturbed NCE were all observed for the *v* (C=O) band of acetone^15^,^19^ in different environment. They attributed the negative NCE to the formation of acetone-metal ion clusters^19^,^20^. Torii H. etc.^15^,^16^,^21^,^22^,^23^,^24^ systematically investigated the Raman noncoincidence effect of acetone, methanol, and N,N-dimethylacetamide etc. with MC or MD computer simulation. Giorgini *et al*.^25^ studied the C=O stretching NCE and the concentration dependence of the acetone solute in dimethylsulphoxide (DMSO) and CCl_4_ respectively. The C=O stretching NCE separation in acetone/DMSO solution exhibited a concave curvature while in acetone/CCl4 solution, exhibited a convex curvature due to the reduction and enhancement of short-range orientational order of acetone respectively^25^.

A great deal of theoretical work has been done on the NCE, most of this is based on the dipole–dipole resonant energy transfer theory. Torii and Tasumi^15^,^21^,^24^ argued that positive NCE phenomenon occurs when the transition dipole is parallel to the permanent dipole, whereas a negative NCE occurs in situation that the transition dipole is head-to-head or parallel side-by-side to the permanent dipole. Wang and McHale^26^ developed a general expression for a coupling Hamiltonian, they concluded that local short range order is not the basic mechanism for NCE. In electrolytic mixtures Giorgini *et al*. proposed the cluster structure and interpreted the negative NCE phenomenon with the results of ab initio quantum chemical calculations^19^,^20^. DFT has been successful in description of vibration spectra of energetic materials, for both single molecules and molecular cluster^27^. Short-range orientational order organization could be treated as cluster and calculated with DFT. In this article we apply DFT calculation for interpretation of acetone NCE phenomenon with optimized aggregated-acetone structure.

To direct observe the existence of aggregated structure of acetone, and trace the dynamic formation of the short-range orientational order organization of acetone molecule, matrix isolation technique was applied. Matrix isolation is a powerful technique for high-resolution spectroscopic study of stable and unstable reaction intermediates as well as weakly bound aggregates^3^,^27^. Using matrix isolation technique, Olbert-Majkut *et al*.^28^ reported the less stable cyclic form of the dimer of acetic acid isolated in argon matrix. NCE phenomenon td-td coupling mechanism predicts that NCE progressively decreases with dilution and eventually vanishes at infinite dilution. In order to obtain the infinite diluted “single acetone molecule” Raman spectra and the dynamic information of td-td coupling “neighborhoods acetone dimer”, the acetone was isolated in argon matrix at high dilution and low temperatures and dynamically photographed upon annealing at different temperature. For the comparison acetone-d6 were conducted for all the Raman and matrix isolation experiment under the same condition.

## Experimental and Computational Methods

### Matrix isolation

Acetone was evaporated to a gas manifold from a glass container maintained at −30 to −20 °C; argon was added up to a total pressure of 800–900 Torr. Acetone was purified by several freezing–annealing cycles in a high-vacuum system. Argon was used without additional purification. Acetone (Aldrich ≥99%) vapor was mixed with argon (Aga, 99.9999%) in a vacuum line and stored in a 2 L pyrex bulb prior to the matrix deposition. The typical acetone:Ar ratio in the gas mixture used for the cryogenic matrix deposition was 3/1000. The mixture was then solidified onto a copper substrate kept at about 6 K within a closed cycle two-stage helium refrigerator (Advanced Research Systems, DE-202SE) equipped with quartz windows. To obtain an optically acceptable matrix, the deposition rate was maintained below 0.11 mmol/min and the total amount of the deposited gas was about 30–60 mmol. The sample temperature was controlled with a Lakeshore 330 temperature controller equipped with a silicon diode.

The 488 nm Raman measurements were carried out on the copper substrate of matrix isolation system with the use of an experimental apparatus consisting of a triple monochromator (TriVista TR557, Princeton Instruments) in subtractive configuration equipped with an argon ion laser (Coherent, CVI MELLES GRIOT) as a source of exciting light at 488 nm (20 mW on the sample) and with a liquid nitrogen cooled CCD array (manufacturer, Princeton Instruments Inc; model ID:LN/2048 × 512.B/I, UVAR.) allowing a wavenumber coverage of 1089 cm^−1^ within the chip active area and a spectral resolution (the instrumental apparatus function, full width at half maxima (FWHM)) of 3 cm^−1^. The accuracy in the measurement of the band positions was 0.5 cm^−1^. The laser beam propagating orthogonally to the sample cell (along the *X* direction in the laboratory frame) was polarized perpendicular to the spectrometer’s optical axis and was focused on the sample with the use of a 60×/0.42 f = 200 objective (S Plan APO HL), which, at the same time, collected the Raman-scattered light in a backscattering geometry. The polarization measurements, performed in the 90° scattering geometry configuration, were carried out in the VV and VH polarization configurations by vertically (V) polarizing the exciting laser light and by alternatively selecting the vertically (V) and horizontally (H) polarized components of the Raman scattered light with the use of a polarization sheet. The polarization measurements were calibrated by checking the depolarization factors of the bands of CCl_4_ at 314 cm^−1^and 459 cm^−1^. In all the runs, we used the same integration time of 100 s and the same accumulations number for all different concentrations to improve the signal-to-noise ratio.

Density functional theory (DFT) calculations were carried out using the hybrid B3LYP-D3 functional to determine the optimized geometry and vibrational frequencies. Complete geometry optimization were performed by using the B3LYP-D3/6-311 G (d,p) level of theory for the molecule of acetone and its dimer and trimer structure, their corresponding frequencies were calculated. All of the DFT calculations made use of the Gaussian program software suite.

## Results and Discussion

**Figure 1:**
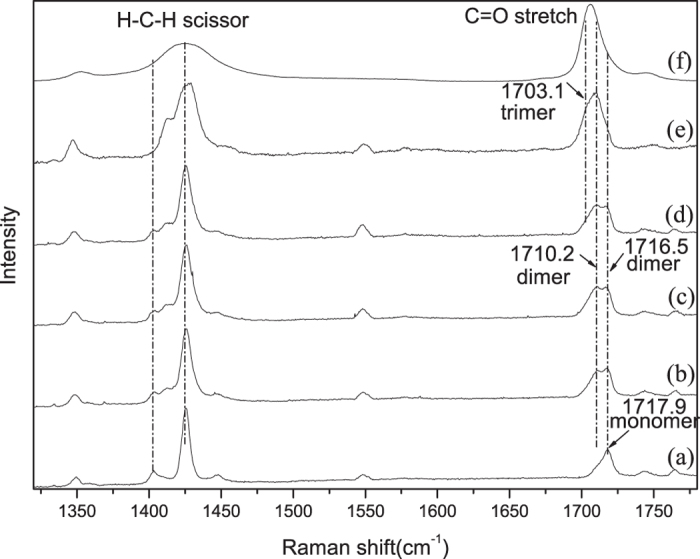
Schematic diagram of reactive monomer for matrix-isolation Raman spectroscopic investigation.

**Figure 2:**
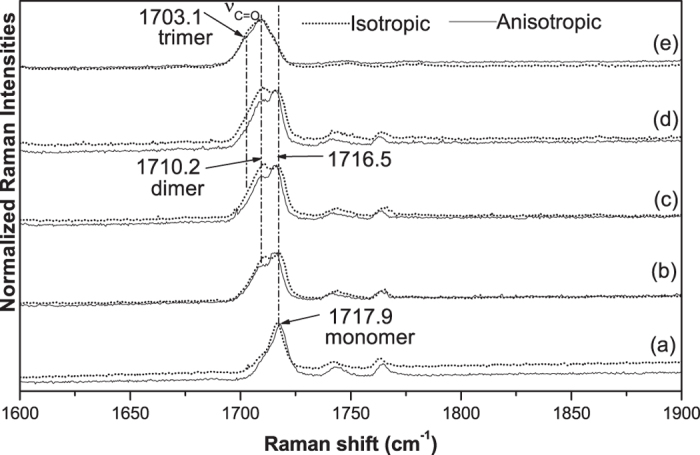
The Raman spectra in the 1320–1780 cm^−1^ regions from co-deposition of CH_3_COCH_3_ in excess Ar. (**a**) 2 h sample deposition at 6k; (**b**–**e**) 16 K, 24 K, 32 K, 45 K annealing respectively. (**f**) 298 K typical collected Raman spectra of CH_3_COCH_3_.

**Figure 3:**
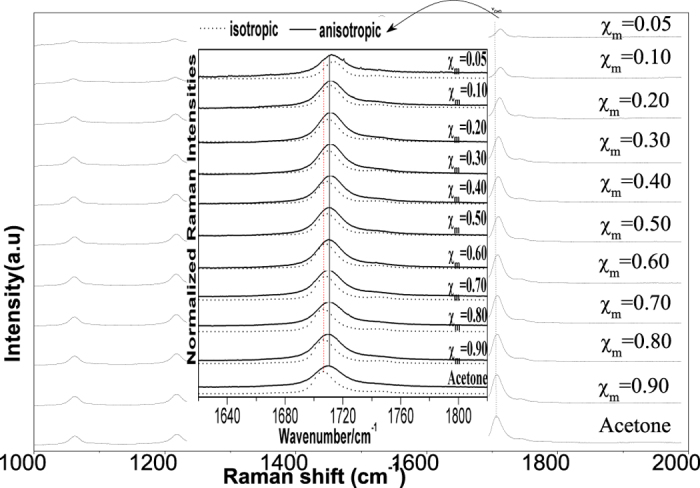
The isotropic and anisotropic Raman spectra in the 1600–1900 cm^−1^ regions from co-deposition of CH_3_COCH_3_ in excess Ar. (**a**) 2 h sample deposition at 6k; (**b**–**e**) 16 K, 24 K, 32 K, 45 K annealing respectively.

The matrix-isolation technique with Raman spectroscopy has proved to be a sensitive and high resolution spectroscopy, it consists of the dispersal of a chemically reactive material in a large excess of an inert solid substance (the matrix) at a temperature low enough to retard or prevent diffusion of the active molecules. A schematic diagram of our reactive monomer for matrix-isolation Raman spectroscopic investigation is shown in Fig. 1. The scarcely isolated monomer were compactly trapped by solid Ar under the same environment, they could be treated as the identical molecules. Figure 2a presents the collected experimental Raman spectra of the copper substrate deposited with 3/1000 acetone/Ar mixtures. The monomer bands are easily distinguished from the aggregated bands, since they disappear or decrease significantly upon the matrix annealing, while bands due to the aggregates become stronger. Additionally, only monomer bands can be observed in the spectra of the freshly deposited matrix with very low acetone/Ar ratio. Thus the frequency at 1717.9 cm^−1^ in Fig. 2a could be assigned as the C=O stretching of the monomer of acetone. After annealing at 16 K (Fig. 2b) the C=O stretching vibration split as two peaks at 1710.2 cm^−1^ and 1716.5 cm^−1^ respectively, these could be assigned to the aggregated dimers of acetone. The C=O stretching relative intensity of peak at 1716.5 cm^−1^ to 1710.2 cm^−1^ decreased with the annealing temperature increased from 16 K to 32 K (Fig. 2c,d). After annealing at 45 K, one weak shoulder peak emerged at 1703.1 cm^−1^, and the relative intensity of peak at 1716.5 cm^−1^ to 1710.2 cm^−1^ continue to decrease to appear as one broad peak (Fig. 2e). We assigned these bands to the trimers of acetone. Figure 3 shows the experimental isotropic and anisotropic Raman spectra of the matrix isolated acetone. To scrutinize the frequency shift the intensity of isotropic and anisotropic Raman spectra have been normalized with the frequency at 1716.5 cm^−1^. Figure 3a–d shows that the isotropic and anisotropic spectra of frequency at 1716.5 cm^−1^ overlap fairly well and only minor intensity difference exists for the frequency at 1710.2 cm^−1^. Isotropic spectra preferentially obtained the intensity of frequency at 1710.2 cm^−1^ while anisotropic spectra preferentially obtained the intensity at 1716.5 cm^−1^. The assignment of the bands is based on our DFT calculation and to certain extent exist discrepancies with previous reported Raman spectra assignment. Nevertheless, some new observations and the support calculation will be discussed below.

**Figure 4:**
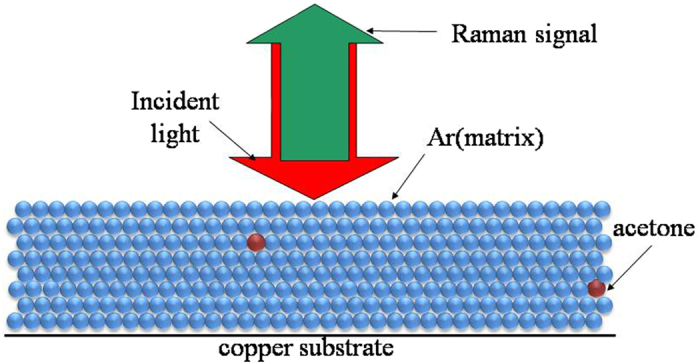
B3LYP-D3/6-311G(d,p) computed geometry parameters of acetone and its aggregates with bond lengths (in angstroms) and bond angles (in degrees) indicated.

We performed full geometry optimization for the acetone monomer, dimer and trimer structure. In order to establish the most stable conformation as the initial point for further calculations, the molecule was submitted to a rigorous conformation analysis around all bonds. We have calculated acetone monomer and two possible geometries of its dimer and trimer at the B3LYP-D3/6-311 G (d,p) level of theory. The two different geometries of the dimer and trimer are shown in Fig. 4, No imaginary vibrational frequencies were found in the further calculation. All DFT calculated vibrational frequencies and ZPE corrected total energy for acetone monomer, dimer and its trimer are given in Table 1. By intermolecular interaction, molecules can form dimers and trimers etc. Dipole-dipole interactions tend to align the molecules to reduce potential energy and increase attraction. According to the energy difference between the dimer or trimer and monomer in Table 1, acetone molecules are more likely to assemble as dimer or trimer. It should be noticed that the two structures are very close in energy. Their energy difference between the dimer b or trimer b and dimer a or trimer a is 12.88 kJ/mol or 24.98 kJ/mol respectively at the B3LYP-D3/6-311 G (d,p) level of theory. As can be seen from Fig. 4 the acetone assembled in a face to face, head-to-tail antiparallel dimeric form through strong intermolecular interaction. Table 1 and Fig. 4 shows that, dimer of two C=O stretching can interact in two extreme ways to form two νC=O vibration modes. One of the ways the C=O stretching interact is *in-phase*, the second way is out-of-phase, which leads to their vibrational frequency and depolarization ratio discrepancy. ***In-phase*** C=O stretching frequency lies below the out-of-phase one. Similarly for the trimer structure, there exist three νC=O vibration modes, one is all C=O stretching *inphase*, the other two are one of the C=O stretching *vibration* out of phase with other two C=O stretching.

The 20 atoms of acetone dimer give rise to 54 normal modes of vibration and trimer give rise to 84 normal modes. The overall 54 normal modes of vibration of this dimer may be considered to be comprised of 48 normal modes arising from these two acetone molecules in-phase and out-of-phase coupling, six modes associated with the relative translation and rotation of two acetone molecules. Similarly 84 mormal modes of trimer comprised of 72 normal modes arising from these three acetone molecules in-phase and out-of-phase coupling, 12 modes associated with the relative translation and rotation of three acetone molecules. Detail description and the comparison of calculated frequencies and corresponding depolarization ratio for monomer, dimer and trimer structures, as well as experimental Raman and IR frequencies are listed in Table 1. Corresponding in-phase and out-of-phase vibration modes may differ in wavenumbers and depolarization ratio, and the magnitude of these splitting will depend on the strength of the interaction between different parts of the neighbor molecules. Table 1 shows that although the aggregated structure of the coupling removes the degeneracy of the C=O stretching vibrational level of the pairs, only few pairs split prominently, but still beyond the resolution limits of the Raman instrument, thus forms a strong broad peak. Thanks to the significant difference in depolarization ratio of C=O stretching pairs, it make possible to collect preferential parallel or perpendicular polarized component Raman spectra, corresponding to isotropic or anisotropic parts by selecting polarization sheet with VV and VH polarization configurations. This parallel or perpendicular polarized spectra preferential collect one component of the C=O stretching pairs, thus separate these two components and lead to the observation of NCE phenomenon. In brief, only those pairs that with prominent vibrational frequency difference and depolarization ratio difference exists NCE effect. Screen the DFT calculation frequency in Table 1, only C=O stretching conforms to these rules, and the Raman spectra NCE phenomenon of acetone also proves this calculation. However, matrix-isolation technique provides alternative method for direct observation of the existence of in-phase and out-of-phase coupled peaks, it rules out the translational and rotational motion thus enhances the spectra resolution.

As have been discussed that in freshly deposited matrix with very low acetone/Ar ratio the acetone could be treated as identical molecules and should be similar with the ideal gas properties. The spectrum in Fig. 2a is corresponding to the DFT calculation monomer freq. listed in Table 1. The strong sharp peak shown at 1717.9 cm^−1^ corresponds to the calculated monomer at 1805 cm^−1^ and can be assigned as C=O stretching vibration. Figure 3a shows the isotropic and anisotroic spectra overlap well (intensity normalized) and no NCE exists, because the acetone presents as monomer structure in this circumstance and there is only one vibration frequency calculated for monomers, it demonstrate that in high dilution there is no NCE phenomenon. With annealing process the argon evaporates gradually, the isolated acetone monomer becomes reactive and collides together to form dimers, accordingly this leads to the split of C=O stretching vibration as two peaks at 1710.2 cm^−1^ and 1716.5 cm^−1^ respectively in the Raman spectra. Relative to the vibration frequency of monomer at 1717.9 cm^−1^, they all red shifted. The split of C=O stretching in the matrix isolation Raman spectra is the direct demonstration for the complex of C=O stretching: it is not a single vibration. These phenomena can be fairly well explained using dimer structure calculated in Table 1. It showed that the calculated frequency of C=O stretching of monomer was higher than both *in-phase* and out-of-phase C=O stretching of dimer. The coupling between the dimers removed the degenerated C=O stretching, resulted in the occurrence of two different vibrational frequency and depolarization ratio. The depolarization ratio of 1716.5 cm^−1^ is higher than another one, this is also in coincidence with the normalized Raman spectra shown in Fig. 3b. The different annealing temperature differ the relative content of dimer in the mixed acetone, the higher temperature the more dimers. Accordingly the Raman relative intensity of isotropic spectra at 1710.2 cm^−1^ get stronger from Fig. 3b to Fig. 3d (Fig. 3 were normalized at 1716.5 cm^−1^) with the enhanced annealing temperature.

One weak shoulder peak appears at 1703.1 cm^−1^ when it anneals at 45 K, this corresponds to the occurrence of trimer structure calculated in Table 1. Again, The DFT calculation of trimer structure in Table 1 shows that, only C=O stretching has prominent vibrational frequency difference and depolarization ratio difference simultaneously, which means the intermolecular interaction among the trimer is mostly through C=O stretching coupling, with other vibration modes degenerated. Compare with the calculated C=O frequency of monomer and dimer, trimera structure red shift more. Three coupled C=O stretching frequency at 1773, 1791 and 1795 cm^−1^ were calculated, which is in consistency with the matrix isolation experiment observation. For comparison, Fig. 2f listed the acetone Raman spectrum measured at 298 K, compared with 45 K annealing spectrum, the C=O vibration red shift more and merge as one strong peak at 298 K. H-C-H scissor at 1425 cm^−1^ also emerged as one broad peak. With the annealing temperature get higher, the trimer b maybe convert to the trimer a. In addition, the patterns of matrix isolation Raman spectra get closer to the 298 K normal Raman spectra.

The experiments were repeated using isotopic labeled acetone-d6 samples. The spectra in selected regions at different annealing temperatures are shown in Figures S1 and S2 of the Supporting Information (SI).

**Figure 5:**
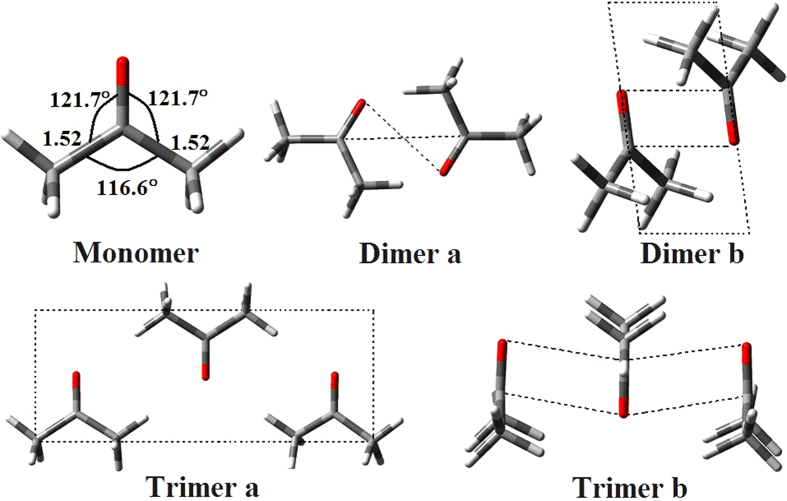
The Raman spectra in the region 1000–2000 cm^−1^ for acetone and ten other volume fractions of acetone, 0.90, 0.80, 0.70, 0.60, 0.50, 0.40, 0.30, 0.20, 0.10 and 0.05 in the binary mixture (CH_3_COCH_3_ + CCl4). Inserted: the ν_c=o_ vibration isotropic and anisotropic parts of the Raman spectra in the region 1680–1760 cm^−1^.

To characterize the concentration effect, we collected the isotropic and anisotropic spectrum in series at a variety of volume fractions of acetone. The Raman spectra in the region of 1000–2000 cm^−1^ for liquid acetone and ten other volume fractions, χ_m_ = 0.90, 0.80, 0.70, 0.60, 0.50, 0.40, 0.30, 0.20, 0.10 and 0.05 of acetone in the binary mixture (CH_3_COCH_3_ + CCl_4_) were shown in Fig. 5. it was found that the Raman frequency of C=O stretching showed a increase in wavenumber with the decrease of acetone concentrations, the νC=O frequencies for liquid acetone and volume fractions of 0.05acetone are 1706.82 cm^−1^ and 1713.04 cm^−1^ respectively. That is to say, from the highest to the lowest concentration of acetone, the wavenumbers of C=O stretching blue shifted about 6.22 cm^−1^, while of other vibrational bands did not shift. Both of the isotropic and anisotropic peak frequencies for C=O stretching mode show an increase in wavenumber with the decrease of solute concentrations, while the value of Δυ_nce_ goes on decreasing upon dilution with CCl_4_ and reduces to the quite low values of 0.50 cm^−1^ at χ_m_ (acetone) = 0.05 for the C=O stretching modes. The FWHM (full width at half maxima) of the C=O stretching modes also get smaller and the peak get sharp with the decrease of solute concentrations.

In general, the Raman shift of the solute molecule in the solutions is determined both by interactions between the solute molecules and by interactions between solute and solvent molecules. When the solute-solute mechanisms dominate, the dilution effect will simply increase the intermolecular distance of the solute molecules and, hence, induce changes in the vibrational wavenumber owing to the weakened interactions. In high concentration, acetone presents in dimer form and the Raman spectra shows broaden and asymmetry peaks, during the dilution process the solute-solvent interaction breaks the dimer to monomer form. The energy difference between monomer and dimer b is 37.92 kJ/mol, easy to be transformed. With the decrease of acetone concentrations the dimer gradually breaks into monomer and the Raman spectra gradually transformed from dimer character to monomer character. That is what we observed in Fig. 5 that the C=O stretching blue shifted and the peak get sharpened and symmetric. The NCE should get smaller and disappeared in extremely diluted solution. Similarly the Supplementary Experiments for isotopic labeled acetone-d6 samples were also conducted. The acetone-d6 Raman spectra in different concentration and corresponding isotropic and anisotropic parts are shown in Figures S3 and S4 of the Supporting Information (SI).

## Conclusion

In summary, the matrix-isolation technique combined with Raman spectroscopy has provided valuable spectroscopic information on monomer acetone spectra and aggregated acetone structure. With the dilution of acetone in carbon tetrachloride, the frequency of C=O stretching mode was observed to blue-shift, while the value of NCE was observed to decrease. In order to explain those phenomena, the isotropic and anisotropic Raman spectra of acetone isolated in an argon matrix at low temperature were collected. It was found that only one sharp C=O vibration band was observed in the spectra of the freshly deposited matrix with very low acetone/Ar ratio. It split into two peaks after annealing at 16 K. The relative intensity of C=O stretching band at 1716.5 cm^−1^ to 1710.2 cm^−1^ decreased with the annealing temperature increased from 16 K to 32 K. After annealing at 45 K, one weak shoulder peak appeared at 1703.1 cm^−1^ while the relative intensity of peak at 1716.5 cm^−1^ to 1710.2 cm^−1^ continue to decrease to the extent that presented as one broad peak. With the enhancement of annealing temperature, the patterns of matrix isolation Raman spectra get closer to the 298 K Raman spectra.

At the same time, the B3LYP-D3/6-311 G (d,p) were performed to calculate monomer, dimer and trimer of acetone, the aggregated structure can well explain the experimental phenomena. The acetone may assemble in a face to face, head-to-tail antiparallel form through strong intermolecular interaction. Experiments and calculation directed at the negative NCE phenomenon are currently in progress.

## Additional Information

**How to cite this article**: Xu, W. *et al*. Study on the noncoincidence effect phenomenon using matrix isolated Raman spectra and the proposed structural organization model of acetone in condense phase. *Sci. Rep.* **7**, 43835; doi: 10.1038/srep43835 (2017).

**Publisher's note:** Springer Nature remains neutral with regard to jurisdictional claims in published maps and institutional affiliations.

## Supplementary Material

Supplementary Dataset 1

## Figures and Tables

**Table 1:**
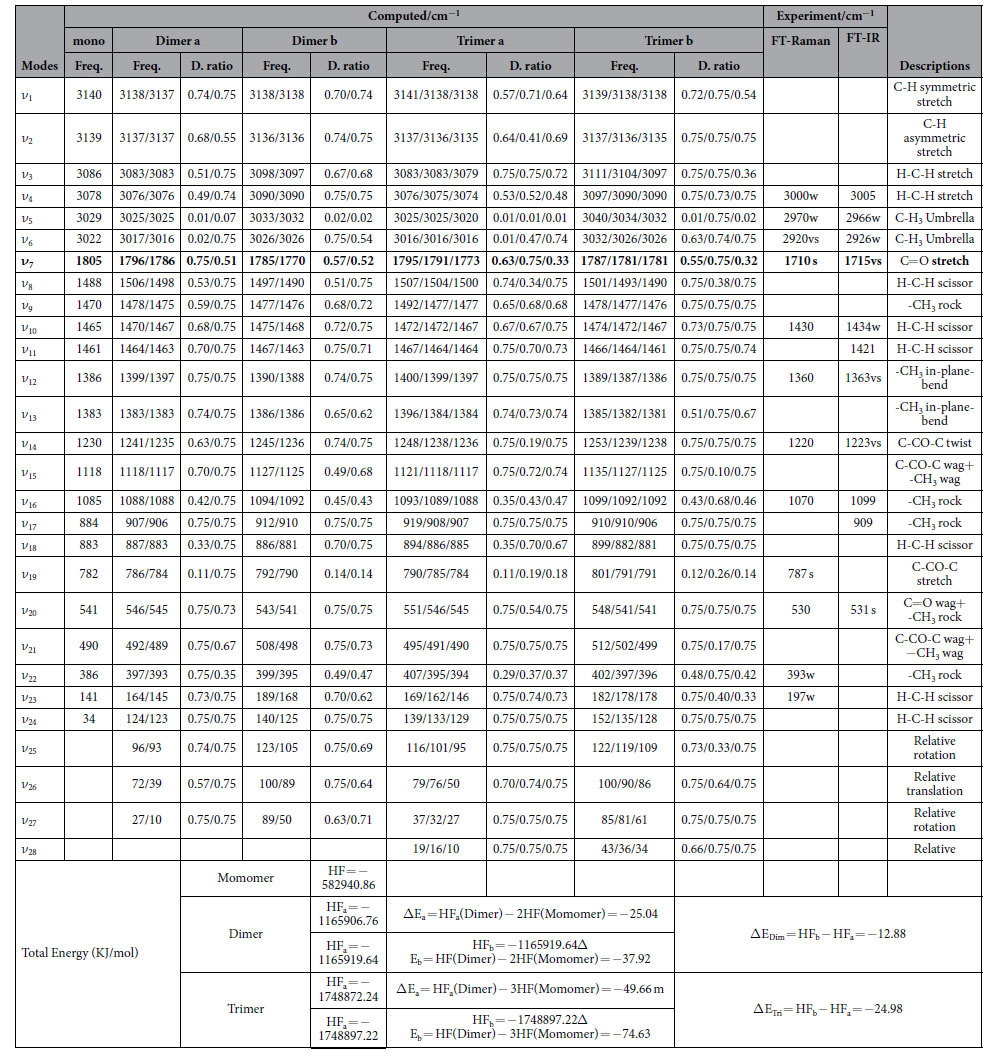
B3LYP-D3/6-311 G(d,p) computed frequency, depolarization ration, ZPE Corrected Energy of acetone monomer, dimer and trimer.
