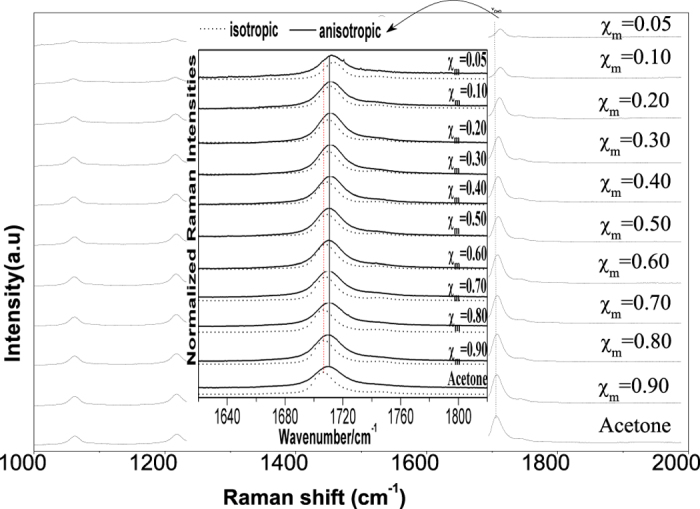# Corrigendum: Study on the noncoincidence effect phenomenon using matrix isolated Raman spectra and the proposed structural organization model of acetone in condense phase

**DOI:** 10.1038/srep46851

**Published:** 2017-07-10

**Authors:** Wenwen Xu, Fengqi Wu, Yanying Zhao, Ran Zhou, Huigang Wang, Xuming Zheng, Bukuo Ni

Scientific Reports 7: Article number: 43835; 10.1038/srep43835 published online: 03 03 2017; updated: 07 10 2017.

This Article contains errors in the order of Figures 1, 2, 3, 4 and 5 which were inadvertently published as Figure 4, 1, 2, 5 and 3 respectively. The correct Figures appear below. The legends for the Figures are correct.

## Figures and Tables

**Figure 1:**
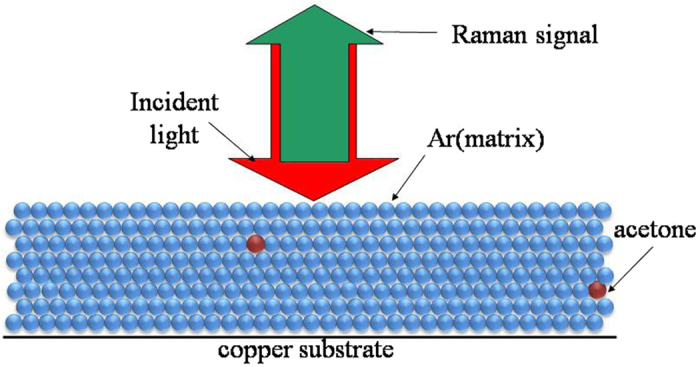


**Figure 2:**
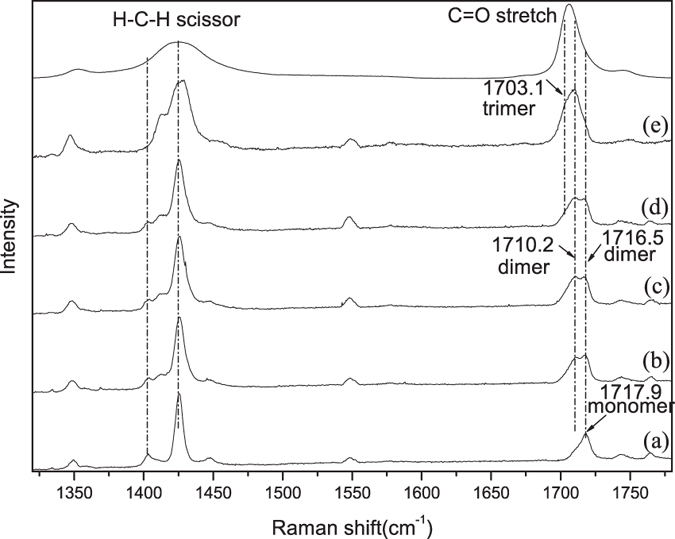


**Figure 3:**
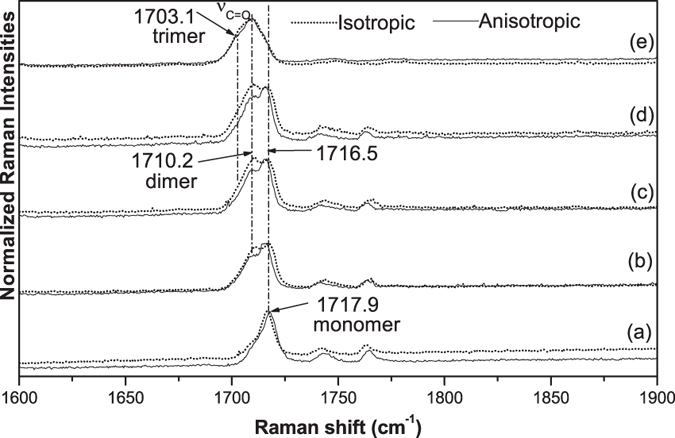


**Figure 4:**
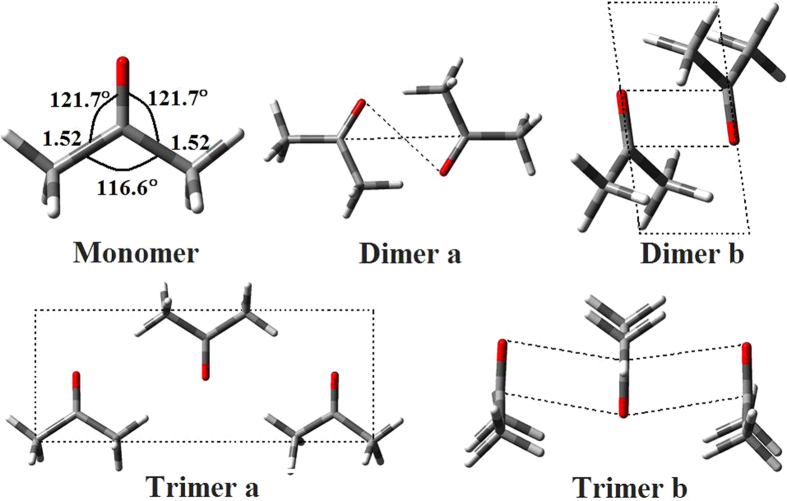


**Figure 5:**