# Supplementary material for: Study on the noncoincidence effect phenomenon using matrix isolated Raman spectra and the proposed structural organization model of acetone in condense phase
Source: Sci Rep. 2017 Mar 3;7:43835. doi: 10.1038/srep43835 (PMC5335557; doi:10.1038/srep43835)
Supplement: Supplementary Dataset 1 [file srep43835-s1.doc]

Supplementary Material

Study on the noncoincidence effect phenomenon using matrix isolated Raman spectra and the proposed structural organization model of acetone in condense phase

*Wenwen Xu^1^, Fengqi Wu^1^, Yanying Zhao^1^, Ran Zhou^1^, Huigang Wang*^1^,Xuming Zheng*^1^,* *Bukuo Ni^2^*

*^1^Department of Chemistry and Engineering Research Center for Eco-dyeing and Finishing of Textiles, MOE, Zhejiang Sci-Tech University, Hangzhou 310018, China*

^2^ *Texas A&M Univ,* *Dept Chem, Commerce,* *TX 75429 USA.*

**Experimental and Computational Methods**

**Matrix isolation** Acetone was evaporated to a gas manifold from a glass container maintained at −30 to −20 ◦C; argon was added up to a total pressure of 800–900 Torr. Acetone was purified by several freezing–annealing cycles in a high-vacuum system. Argon was used without additional purification. Acetone (Aldrich ≥ 99%) vapor was mixed with argon (Aga, 99.9999%) in a vacuum line and stored in a 2L pyrex bulb prior to the matrix deposition. The typical acetone:Ar ratio in the gas mixture used for the cryogenic matrix deposition was 3/1000.The mixture was then solidified onto a copper substrate kept at about 6 K within a closed cycle two-stage helium refrigerator (Advanced Research Systems, DE-202SE) equipped with quartz windows. To obtain an optically acceptable matrix, the deposition rate was maintained below 0.11 mmol/min and the total amount of the deposited gas was about 30–60 mmol. The sample temperature was controlled with a Lakeshore 330 temperature controller equipped with a silicon diode.

The 488nm Raman measurements were carried out on the copper substrate of matrix isolation system with the use of an experimental apparatus consisting of a triple monochromator (TriVista TR557, Princeton Instruments) in subtractive configuration equipped with an argon ion laser(Coherent, CVI MELLES GRIOT)as a source of exciting light at 488nm (20mW on the sample) and with a liquid nitrogen cooled CCD array (manufacturer, Princeton Instruments Inc; model ID:LN/2048X512.B/I,UVAR.)allowing a wavenumber coverage of 1089 cm^-1^ within the chip active area and a spectral resolution (the instrumental apparatus function, full width at half maxima (FWHM)) of 3 cm^-1^.The accuracy in the measurement of the band positions was 0.5 cm^-1^.The laser beam propagating orthogonally to the sample cell(along the *X* direction in the laboratory frame) was polarized perpendicular to the spectrometer’s optical axis and was focused on the sample with the use of a 60×/0.42 f=200objective (S Plan APO HL),which, at the same time, collected the Raman-scattered light in a backscattering geometry. The polarization measurements, performed in the 90°scattering geometry configuration, were carried out in the VV and VH polarization configurations by vertically (V) polarizing the exciting laser light and by alternatively selecting the vertically (V) and horizontally (H) polarized components of the Raman scattered light with the use of a polarization sheet. The polarization measurements were calibrated by checking the depolarization factors of the bands of CCl_4_ at 314 cm^-1^and 459 cm^-1^. In all the runs, we used the same integration time of 100 s and the same accumulations number for all different concentrations to improve the signal-to-noise ratio.

Density functional theory (DFT) calculations were carried out using the hybrid B3LYP-D3 functional to determine the optimized geometry and vibrational frequencies. Complete geometry optimization were performed by using the B3LYP-D3/6-311+G (d,p) level of theory for the molecule of acetone and its dimer and trimer structure were also optimized and their frequencies were included. All of the DFT calculations made use of the Gaussian program software suite.

FigureS1 The Raman spectra in the 1600-1800 cm^-1^ regions from co-deposition of CD_3_COCD_3_ in excess Ar. (a) 2h sample deposition at 6k; (b)-(e) 15K, 25K, 35K, 45K annealing respectively.(f) 298K typical collected Raman spectra of CD_3_COCD_3_

Figure S2 The isotropic(red curve) and anisotropic(black curve) Raman spectra in the 1600-1800 cm^-1^ regions from co-deposition of CD_3_COCD_3_ in excess Ar. (a) 2h sample deposition at 8k; (b)-(e) 15K, 25K, 35K, 45K annealing respectively.

Figure S3 The concentration dependent ofν_c=o_ vibration ofthe Raman spectra in the region 1680–1750 cm^-1^ for CD_3_COCD_3_ and ten other volume fractions of CD_3_COCD_3_, 0.90, 0.80, 0.70, 0.60, 0.50, 0.40 and 0.30 in the binary mixture(CD_3_COCD_3_+CCl_4_) .

Figure s4 The ν_c=o_ vibration isotropic(red curve) and anisotropic(black curve) parts of the Raman spectra in the region 1680–1750 cm^-1^ for CD_3_COCD_3_ and ten other volume fractions of CD_3_COCD_3_, 0.90, 0.80, 0.70, 0.60, 0.50, 0.40 and 0.30 in the binary mixture(CD_3_COCD_3_+CCl_4_) .
